# Effect of Fish Oil Supplementation on Fasting Vascular Endothelial Function in Humans: A Meta-Analysis of Randomized Controlled Trials

**DOI:** 10.1371/journal.pone.0046028

**Published:** 2012-09-21

**Authors:** Wei Xin, Wei Wei, Xiaoying Li

**Affiliations:** 1 First Department of Geriatric Cardiology, Chinese PLA General Hospital, Beijing, PR China; 2 Medical College of Nankai University, Tianjin, PR China; Medical University Innsbruck, Austria

## Abstract

**Background:**

Effect of fish oil supplementation on flow-mediated dilation, an index of endothelial function in humans, remains controversial. We performed a meta-analysis to determine whether fish oil supplementation could improve endothelial function.

**Methods:**

Human intervention studies were identified by systematic searches of Medline, Embase, Cochrane's library and references of related reviews and studies. A random-effect model was applied to estimate the pooled results. Meta-regression and subgroup analyses were performed to evaluate the impact of study characteristics on the effect of fish oil supplementation on flow-mediated dilation.

**Results:**

A total of sixteen records with 1,385 subjects were reviewed. The results of the pooled analysis showed that fish oil supplementation significantly improved flow-mediated dilation (weighed mean difference: 1.49%, 95% confidence interval 0.48% to 2.50%, p = 0.004). Meta-regression and subgroup analysis suggested that the quality of included studies were inversely related to the overall effect (regression coefficient  = −1.60, p = 0.04), and the significance of the effect was mainly driven by the studies with relatively poor quality. Sensitivity analysis including only double-blind, placebo-controlled studies indicated fish oil supplementation has no significant effect on endothelial function (weighed mean difference: 0.54%, 95% confidence interval −0.25% to 1.33%, p = 0.18). Besides, normoglycemic subjects or participants with lower diastolic blood pressure seemed to be associated with remarkable improvement of endothelial function after fish oil supplementation.

**Conclusions:**

Although current evidence suggested a possible role of fish oil in improving endothelial function, large-scale and high-quality clinical trials are needed to evaluate these effects before we can come to a definite conclusion.

## Introduction

Accumulating evidence from epidemiological studies and clinical trials have suggested that increased intake of non-fried fish or supplementation with fish oil is associated with lower risk of cardiovascular mortality, indicating a potential role of fish oil supplementation in the primary and secondary prevention of cardiovascular diseases (CVD) [1], [2]. Fish oil – mainly consisting of two categories of marine omega 3 polyunsaturated fatty acids (PUFAs) – eicosapentaenoic acid (EPA) and docosahexaenoic acid (DHA) – may exert its cardioprotective effects via many mechanisms including lowering blood pressure, regulation of blood lipids, lowering heart rate, anti-inflammation, anti-arrhythmia, and possible improvement of endothelial dysfunction et al. [3], [4].

Endothelial dysfunction is considered to be an early pathophysiologic feature of many CVD including atherosclerosis and hypertension et al., and is also an independent predictor and prognostic factor for these CVD [5]–[8]. Clinically, endothelial function can be determined by measuring flow-mediated dilation (FMD), which represents the ability of the brachial artery to dilate in response to ischemia-induced hyperemia and reflects of the local bioavailability of endothelium-derived vasodilator, mainly including nitric oxide (NO) [9]–[11]. Although a cross-sectional study has suggested that dietary fish or fish oil consumption may be associated with enhanced FMD in women [12], results of prospective randomized controlled trials evaluating the effect of fish oil supplementation on FMD, a surrogate of endothelial function, are generally controversial [13]–[28], partly due to the small number of included participants. Therefore, we performed a meta-analysis to systematically evaluate the effect of fish oil supplementation on FMD in humans. More importantly, we tried to explore the influence of the participant and study characteristics, especially those of established risk factors, to the effect of fish oil on endothelial function.

## Methods

This systematic review and meta-analysis was performed according to PRISMA (Preferred Reporting Items for Systematic Reviews and Meta-Analyses) statement [29] and Cochrane Handbook guidelines [30].

**Figure 1:**
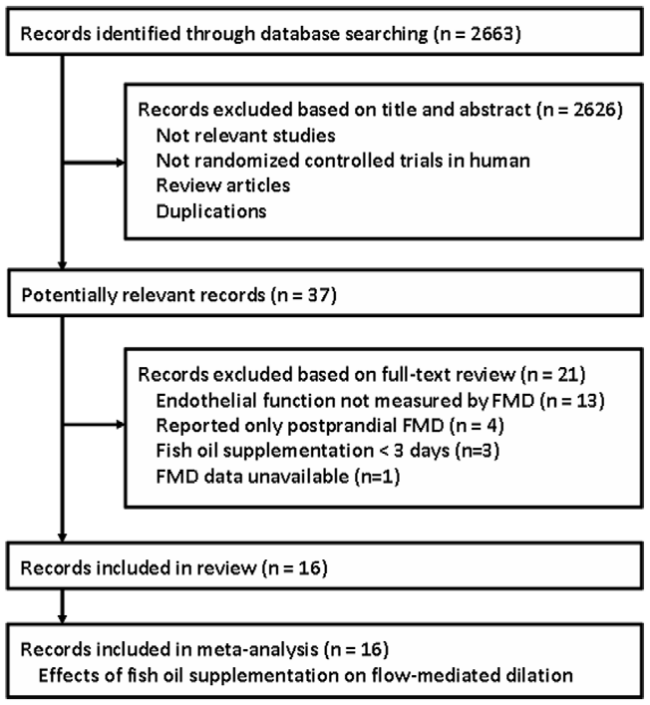
Flow diagram of the study selection procedure.

### Search strategy

We systematically searched Pubmed (from 1950 to February, 2012), Embase (from 1966 to February, 2012) and the Cochrane Library (Cochrane Center Register of Controlled Trials) for relevant records, using the term “omega-3 fatty acids”, “fish oil”, “fish-oil”, “marine oil”, “eicosapentaenoic acid”, “EPA”, “docosahexaenoic acid”, “DHA”, “dietary therapy” paired with the following: “endothelial”, “endothelium”, “coronary heart disease”, “cardiovascular disease”, “stroke”, “cerebrovascular disease”, and “trial”. The search was limited to studies in humans. We also analyzed reference lists of original and review articles using a manual approach.

**Table 1:** Baseline characteristics of participants of included studies.

Study	Participants	No. of subjects	Mean age	Male	BMI	T2DM	Smokers	Mean SBP	Mean DBP	Mean TG	Mean TC	Statins used	ACEI/ARB used	Baseline FMD
			years	%	kg/m^2^	%	%	mmHg	mmHg	mmol/L	mmol/L			%
**Woodman 2003a**	Hypertensive T2DM	20	61.3	78.8	28.9	100	0	130.6	71.5	1.49	4.55	Partially	Partially	3.26
**Woodman 2003b**	Hypertensive T2DM	18	61.2	73.5	30.3	100	0	130.6	70.5	1.65	4.55	Partially	Partially	4.5
**Dyerberg 2004**	Healthy males	50	38.4	100	24.5	0	21.6	127.5	77.6	1.18	4.95	None	None	3.75
**Engler 2004**	Dyslipidemic children	20	14	NR	21	0	0	115	58	1.57	7.33	NR	NR	5.9
**Prabodh 2007**	Healthy adults	26	31	65.4	23.4	0	0	113.2	64.3	1.24	NR	None	None	NR
**Hill 2007a**	Overweight adults	65	50.3	36.9	34.1	0	0	130.1	74.5	1.79	5.91	None	None	4.25
**Hill 2007b**	Overweight adults	65	50.3	36.9	34.1	0	0	130.1	74.5	1.79	5.91	None	None	4.25
**Schiano 2008**	PAD	32	66	90.6	26.9	46.9	94	NR	NR	1.77	5.1	Partially	Partially	6.9
**Mindrescu 2008**	Dyslipidemic South Asians	30	51	76.7	27.1	33.3	NR	123.3	73.1	1.56	4.87	All	Partially	−1.99
**Wright 2008a**	SLE	56	48.1	6.7	25.6	0	15	125.5	72.4	1.05	4.8	None	None	2.74
**Wright 2008b**	SLE	56	48.1	6.7	25.6	0	15	125.5	72.4	1.05	4.8	None	None	2.74
**Rizza 2009**	Normoglycemic offspring of T2DM	50	29.9	50	26.2	0	NR	115.1	76.9	1.33	4.99	None	None	7.9
**Stirban 2010**	T2DM	32	56.8	NR	31.2	100	NR	139	81	1.6	4.84	Partially	Partially	5.54
**Wong 2010**	T2DM	97	60.1	44.3	25.8	100	17.5	137.5	78	1.45	4.9	Partially	Partially	3
**Hileman 2011**	HIV-infected adults	31	51	100	25	0	74.3	118.5	79	1.4	4.64	None	None	3.13
**Haberka 2011**	AMI, successful PCI	40	60±9	80	28.9	20	52.5	NR	NR	1.59	5.05	All	All	9.5
**Skulas-Ray 2011a**	Hypertriglyceridemic	26	44.3	88.5	29	0	0	122.9	81.9	2.52	5.36	None	None	5
**Skulas-Ray 2011b**	Hypertriglyceridemic	26	44.3	88.5	29	0	0	122.9	81.9	2.52	5.36	None	None	5
**Sanders 2011a**	Nonsmokers	151	55	38.5	25.9	0	0	120.5	77	1.15	5.45	Partially	Partially	5.39
**Sanders 2011b**	Nonsmokers	150	55	38.7	26.4	0	0	121	77.5	1.14	5.45	Partially	Partially	4.85
**Sanders 2011c**	Nonsmokers	151	55	38.9	25.9	0	0	120.5	76.5	1.13	5.45	Partially	Partially	5.01
**Moertl 2011a**	Nonischemic CHF	30	57.3	80	27.5	16.7	NR	110.5	72.9	NR	4.43	NR	All	8.41
**Moertl 2011b**	Nonischemic CHF	29	59.3	86.2	27.9	24.1	NR	110.3	74	NR	4.46	NR	All	8.46

The studies by woodman (2003), Skulas-Ray (2011) and Moertl (2011) include two intervention groups with different fish oil doses separately, and the study by Sanders (2011) includes three intervention groups with different fish oil doses. The studies by Hill (2007) and Wright (2008) each contain two comparisons with different fish oil treatment durations.

BMI, body mass index; SBP, systolic blood pressure; DBP, diastolic blood pressure; TG, triglyceride; TC, total cholesterol; ACEI, angiotensin-converting enzyme inhibitor; ARB, angiotensin receptor blocker; FMD, flow-mediated dilation; T2DM, type 2 diabetes mellitus; PAD, peripheral artery disease; SLE, systemic lupus erythematosus; HIV, human immunodeficiency virus; AMI, acute myocardial infarction; PCI, percutaneous coronary intervention; CHF, chronic heart failure; NR, not reported.

**Table 2:** Characteristics of study design of included studies.

Study	Study design	Fish oil dose	DHA dose	EPA dose	Control	Duration	Occlusion position	Occlusion duration	Jadad Score
		mg/d	mg/d	mg/d		weeks		min	
**Woodman 2003a**	R, PC, DB	4000	0	4000	Olive oil	6	forearm	5	3
**Woodman 2003b**	R, PC, DB	4000	4000	0	Olive oil	6	forearm	5	3
**Dyerberg 2004**	R, PC, DB	3168	1320	1848	Palm oil	8	forearm	4.5	4
**Engler 2004**	R, PC, DB, CO	1200	1200	0	Corn/soy oil	6	forearm	5	3
**Prabodh 2007**	R, PC, SB	500	200	300	Corn oil	2	forearm	3	3
**Hill 2007a**	R, PC, DB	1920	1560	360	Sunflower oil	6	forearm	5	3
**Hill 2007b**	R, PC, DB	1920	1560	360	Sunflower oil	12	forearm	5	3
**Schiano 2008**	R, SB	1700	1063	637	No treatment	13	forearm	5	2
**Mindrescu 2008**	R, CO	4530	1950	2580	No treatment	4	forearm	5	2
**Wright 2008a**	R, PC, DB	3000	1200	1800	Olive oil	12	forearm	4.5	4
**Wright 2008b**	R, PC, DB	3000	1200	1800	Olive oil	24	forearm	4.5	4
**Rizza 2009**	R, PC, DB	1700	1020	680	Olive oil	12	upper arm	5	3
**Stirban 2010**	R, PC, DB, CO	1680	760	920	Olive oil	6	forearm	4.5	4
**Wong 2010**	R, PC, DB	2680	1000	1680	Olive oil	12	forearm	5	5
**Hileman 2011**	R, PC, DB	1660	730	930	Olive oil	24	forearm	5	4
**Haberka 2011**	R, SB	840	375	465	No treatment	4	forearm	3	2
**Skulas-Ray 2011a**	R, PC, DB, CO	840	375	465	Corn oil	8	forearm	5	3
**Skulas-Ray 2011b**	R, PC, DB, CO	3360	1500	1860	Corn oil	8	forearm	5	3
**Sanders 2011a**	R, PC, DB	450	180	270	Olive oil	52	forearm	5	5
**Sanders 2011b**	R, PC, DB	900	360	540	Olive oil	52	forearm	5	5
**Sanders 2011c**	R, PC, DB	1800	720	1080	Olive oil	52	forearm	5	5
**Moertl 2011a**	R, PC, DB	840	375	465	gelatin	13	upper arm	5	5
**Moertl 2011b**	R, PC, DB	3360	1500	1860	gelatin	13	upper arm	5	5

The studies by woodman (2003), Skulas-Ray (2011) and Moertl (2011) include two intervention groups with different fish oil doses separately, and the study by Sanders (2011) includes three intervention groups with different fish oil doses. The studies by Hill (2007) and Wright (2008) each contain two comparisons with different fish oil treatment durations.

DHA, docosahexaenoic acid; EPA, eicosapentaenoic acid; R, randomized; PC, placebo-controlled; DB, double-blinded; SB, single-blinded; CO, crossover.

### Study selection

Original studies were included if they met the following criteria: 1) published as full-length articles in English; 2) reported as a prospective, randomized, and controlled trial with either a parallel or a crossover design (regardless of sample size); 3) analyzed human subjects who were assigned to oral fish oil supplementation or a control group for ≥3 days; 4) evaluated endothelial function by measuring of fasting FMD in the brachial artery; 5) data [means and standard deviations (SDs)] concerning changes in FMD from baseline were reported or could be estimated. Review articles, nonhuman studies, observational studies without longitudinal follow-up, cross-sectional studies, duplicate publications, and studies in which changes in FMD were not reported or could not be estimated were excluded.

**Figure 2:**
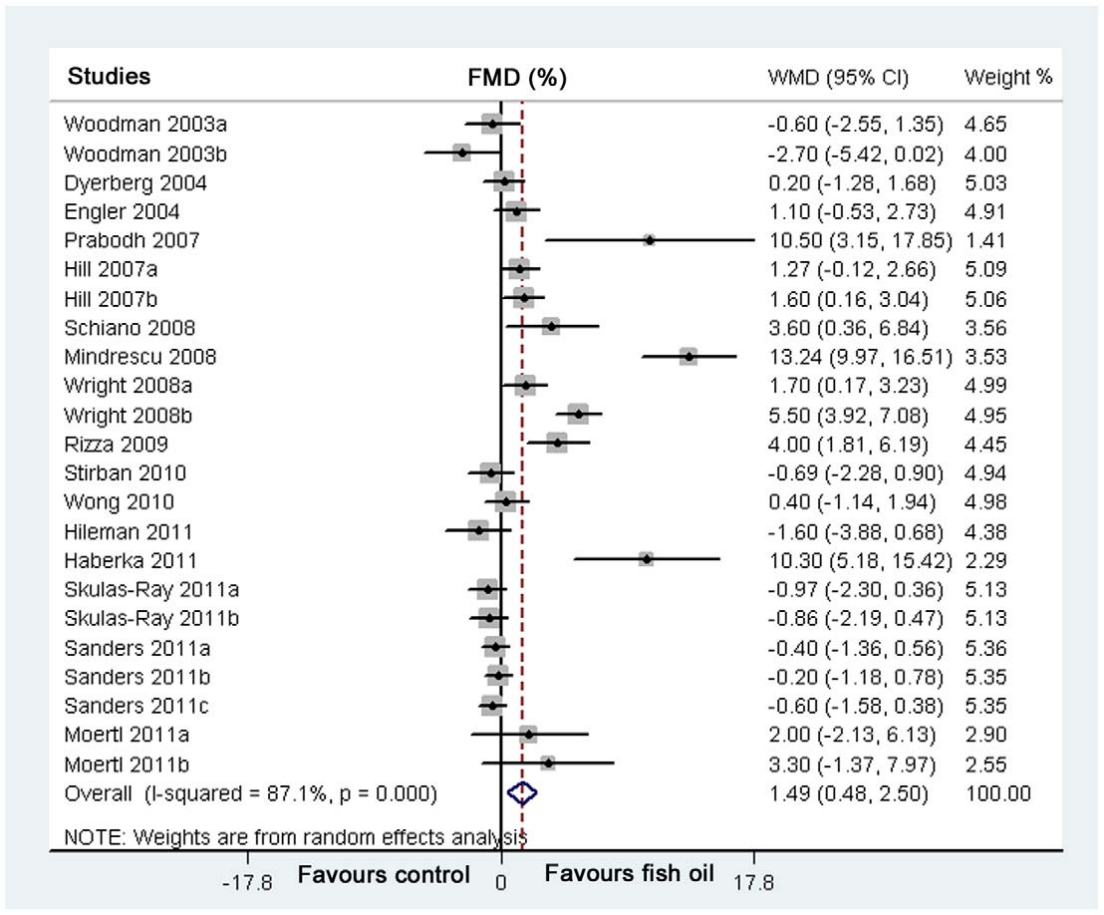
Forest plots from meta-analysis of weighed mean difference in flow-mediated dilation for subjects randomized to fish oil or control groups. The effect size of each study is proportional to the statistical weight. The diamond indicates the overall summary estimate for the analysis; the width of the diamond represents the 95% CI. FMD, flow-mediated dialtion; WMD, weighed mean difference; CI, confidence interval.

### Data extraction and quality assessment

Two authors (WX and WW) independently performed the literature searching, data extraction, and quality assessment according to inclusion criteria. Discrepancies were resolved by consensus. Extracted data included study design characteristics (parallel or crossover), patient characteristics [e.g., number, age, sex, body mass index (BMI), general healthy status, percentages of the smokers and patients with type 2 diabetes (T2DM), baseline systolic blood pressure (SBP), diastolic blood pressure (DBP), serum triglyceride (TG) and total cholesterol (TC) levels, usage of statins, angiotensin-converting enzyme inhibitors (ACEIs) or angiotensin receptor blockers (ARBs), and baseline FMD of the participants], intervention strategies (total dose of fish oil, dose of EPA and DHA, and the treatment in control groups), follow-up duration, and means and SDs for changes of FMD from baseline. Because previous studies indicated that technical aspects of FMD measurement may influence the results [10], characteristics of FMD measurement (occlusion position and occlusion duration) were also extracted for analysis. If these data were missing or not reported in the content of the paper, corresponding authors were contacted to ask if the unpublished data were available. For trials in which fish oil was supplied of more than one dose or treatment duration (e.g., FMD data were repeatedly measured at more than one time point) multiple studies were considered.

**Table 3:** Characteristics associated with net change in flow-mediated dilation: univariate meta-regression analysis.

	FMD (%)		
	Coefficient	95% CI	p
Number of subjects	−0.017	−0.056 to 0.021	0.36
Mean age (years)	−0.03	−0.18 to 0.11	0.65
Males' percentage (%)	−0.006	−0.071 to 0.059	0.85
BMI (kg/m^2^)	−0.15	−0.70 to 0.39	0.39
Smokers' percentage (%)	0.029	−0.027 to 0.084	0.29
SBP (mmHg)	−0.12	−0.33 to 0.09	0.26
DBP (mmHg)	−0.20	−0.49 to 0.09	0.14
Percentage of patients with T2DM (%)	−0.017	−0.036 to 0.002	0.10
Mean TG (mmol/L)	−1.46	−5.92 to 3.01	0.50
Mean TC (mmol/L)	−0.47	−3.03 to 2.08	0.70
Mean baseline FMD (%)	−0.14	−0.43 to 0.15	0.33
Fish oil dose (mg/d)	0.0003	−0.0011 to 0.0018	0.67
DHA dose (mg/d)	−0.0001	−0.0022 to 0.0020	0.92
EPA dose (mg/d)	0.0005	−0.0012 to 0.0023	0.54
Duration (weeks)	−0.06	−0.17 to 0.04	0.21
Jadad Score	−1.60	−3.13 to −0.08	0.04

FMD, flow-mediated dilation; CI, confidence interval; BMI, body mass index; SBP, systolic blood pressure; DBP, diastolic blood pressure; T2DM, type 2 diabetes; TG, triglyceride; TC, total cholesterol; DHA, docosahexaenoic acid; EPA, eicosapentaenoic acid.

The quality of the studies was judged by Jadad Score, which evaluates the quality of randomization, generation of random numbers, concealment of treatment allocation, blinding, and reporting of withdrawals [31]. Trials scored one point for each area addressed, with a possible score between 0 and 5, where 5 represented the highest level of quality.

**Table 4:** Subgroup analyses for the effect of fish oil supplementation on flow-mediated dilation according to predefined study characteristics.

Study characteristics	FMD (%)				
	Studies (patients), n	I^2^	WMD [95% CI]	p value for subgroup effects	p value for subgroup interaction
**Number of subjects**					
<40	12 (454)	88%	1.53 [−0.33, 3.39]	0.11	0.96
≥40	11 (931)	87%	1.58 [0.40, 2.77]	0.009	
**Health status**					
Generally healthy	6 (578)	78%	0.61 [−0.63, 1.86]	0.34	0.22
Chronic condition	17 (807)	88%	1.76 [0.39, 3.14]	0.01	
**Mean age**					
<51 years	10 (512)	86%	1.70 [0.32, 3.07]	0.02	0.72
≥51 years	13(873)	87%	1.33 [−0.11, 2.77]	0.07	
**Percentage of males**					
≤50%	9 (841)	87%	1.36 [0.14, 2.58]	0.03	0.44
>50%	12 (440)	89%	2.33 [0.21, 4.45]	0.03	
**Mean BMI**					
<27 kg/m^2^	12 (890)	85%	1.32 [0.14, 2.50]	0.03	0.65
≥27 kg/m^2^	11 (495)	89%	1.83 [−0.04, 3.70]	0.06	
**Diabetic status**					
Normoglycemic	14 (995)	85%	0.92 [−0.07, 1.91]	0.07	0.04
Diabetic	4 (199)	23%	−0.58 [−1.63, 0.47]	0.28	
**Mean SBP**					
<122 mmHg	9 (658)	73%	0.72 [−0.43, 1.87]	0.22	0.53
≥122 mmHg	12 (655)	91%	1.33 [−0.21, 2.86]	0.09	
**Mean DBP**					
<75 mmHg	11 (465)	89%	2.84 [0.90, 4.78]	0.004	0.01
≥75 mmHg	10 (848)	54%	−0.21 [−0.85, 0.42]	0.51	
**Mean TG**					
<1.5 mmol/L	11 (836)	87%	0.91 [−0.39, 2.20]	0.17	0.27
≥1.5 mmol/L	11 (490)	90%	2.17 [0.35, 4.00]	0.02	
**Mean TC**					
≤4.95 mmol/L	11 (511)	91%	1.73 [−0.29, 3.74]	0.09	0.44
>4.95 mmol/L	11 (848)	79%	0.85 [−0.11, 1.82]	0.08	
**Statins used**					
All or partially	10 (783)	90%	1.49 [−0.15, 3.13]	0.07	0.99
None	10 (503)	87%	1.48 [−0.00, 2.95]	0.05	
**ACEIs/ARBs used**					
All or partially	12 (842)	88%	1.61 [0.08, 3.14]	0.04	0.90
None	10 (503)	87%	1.48 [−0.00, 2.95]	0.05	
**Baseline FMD**					
<5%	11 (668)	91%	1.55 [−0.07, 3.17]	0.06	0.54
≥5%	11 (691)	77%	0.92 [−0.22, 2.06]	0.11	
**Fish oil dose**					
<1800 mg/d	11 (666)	80%	1.17 [−0.10, 2.45]	0.07	
≥1800 mg/d	12 (719)	91%	1.63 [0.08, 3.18]	0.04	0.65
**DHA dose**					
≤1100 mg/d	13 (894)	76%	0.62 [−0.38, 1.62]	0.22	0.14
>1100 mg/d	10 (491)	91%	2.22 [0.37, 4.07]	0.02	
**EPA dose**					
≤640 mg/d	11 (669)	77%	1.01 [−0.15, 2.18]	0.09	0.48
>640 mg/d	12 (716)	91%	1.75 [0.09, 3.41]	0.04	
**Duration**					
<12 weeks	11 (487)	90%	1.83 [−0.00, 3.65]	0.05	0.70
≥12 weeks	12 (898)	85%	1.39 [0.20, 2.58]	0.02	
**Jadad Score**					
2 points	3 (132)	88%	8.99 [2.59, 15.40]	0.006	0.02
3 points	9 (388)	78%	0.65 [−0.63, 1.94]	0.32	
4 points	5 (257)	90%	1.07 [−1.32, 3.46]	0.38	
5 points	6 (608)	0%	−0.22 [−0.75, 0.30]	0.40	
**Occlusion position**					
Forearm	20 (1276)	88%	1.29 [0.24, 2.34]	0.02	–
**Occlusion duration**					
4.5∼5 min	21 (1319)	87%	1.13 [0.15, 2.10]	0.02	–
**Study design**					
R, DB, PC	19 (1227)	79%	0.54 [−0.25, 1.33]	0.18	–

FMD, flow-mediated dilation; BMI, body mass index; SBP, systolic blood pressure; DBP, diastolic blood pressure; TG, triglyceride; TC, total cholesterol; ACEI, angiotensin-converting enzyme inhibitor; ARB, angiotensin receptor blocker; DHA, docosahexaenoic acid; EPA, eicosapentaenoic acid; R, randomized; DB, double-blinded; PC, placebo-controlled; WMD, weighed mean difference; CI, confidence interval.

**Figure 3:**
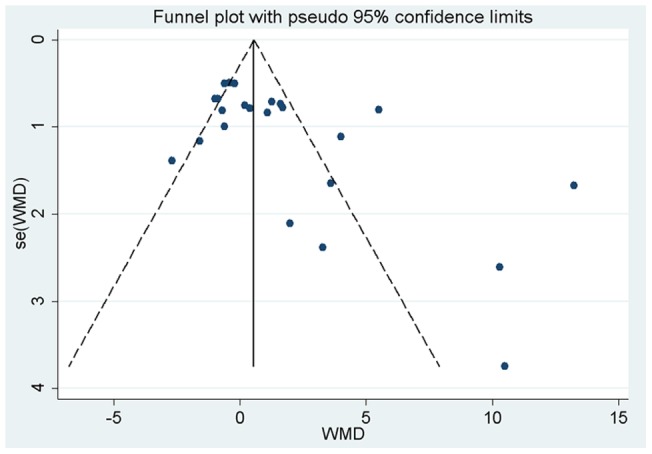
Funnel plots (with pseudo 95% CIs) of all individual studies in the meta-analyses of standard deviation of flow-mediated dilation. FMD, flow-mediated dialtion; WMD, weighed mean difference; CI, confidence interval.

### Statistical analysis

The primary outcome of this meta-analysis was the change in FMD between baseline and endpoint in response to oral fish oil intervention. The pooled effect was presented as weighted mean difference (WMD) with 95% confidence intervals (CI). Inter-study heterogeneity was formally tested using Cochrane's test, and significant heterogeneity was considered existing if p value was <0.10. The I^2^ statistic, which describes the percentage of total variation across studies that is due to heterogeneity rather than chance, was also examined, and a value of I^2^>50% indicated significant heterogeneity [32]. We used a random-effect model to estimate the overall effect instead of a fixed-effect model, considering that this is a more conservative method that takes into account that study heterogeneity can vary beyond chance, thus providing a more generalizable results. Univariate meta-regression analysis was performed to identify the possible source of heterogeneity, using the following variables: number of subjects in each study; mean age; percentage of males; BMI; percentage of smokers; SBP, DBP, percentage of patients with T2DM; mean TG level; mean TC level; baseline FMD; fish oil dose; DHA dose; EPA dose; follow-up duration and Jadad Score. Predefined subgroup analyses were also performed to further explore the possible influence of the above study characteristics on the pooled outcome. Median values of continuous variables were used as cutoff values for grouping studies. Sensitivity analysis by excluding certain studies was performed to test the stability of the results. Furthermore, potential publication bias was assessed with funnel plot, Egger regression asymmetry test [33], as well as fail-safe N test [30]; p values were two-tailed and statistical significance was set at 0.05. Meta-analysis and statistical analysis was performed with Stata software (version 12.0; Stata Corporation, College Station, TX).

## Results

### Search results

A total of 2663 records were identified, and 2626 were excluded because they were review articles or duplications, did not describe randomization or controlling, or because the objectives of these studies were irrelevant to the present meta-analysis. Of the 37 potentially relevant records screened, sixteen [13]–[28] met the selection criteria for the current meta-analysis (**Figure** **1**). Twenty-one records were excluded because endothelial function was not measured by FMD in 13 records; only data of postprandial FMD were reported in 4 records; fish oil was supplemented for <3 days in 3 records; and because fasting FMD data were unavailable in one record.

### Study characteristics

Overall, a total of 23 studies from 16 published articles were included in the current meta-analysis, which comprised a total of 1385 participants, 707 subjects in the fish oil group and 678 patients in the control group. The characteristics of these participants and the studies were shown in **Table** **1** and **Table** **2**. All of the included studies were prospective randomized controlled trials; of which nineteen studies [13], [14], [16]–[21], [23]–[25], [27], [28] were of parallel-design and the other four [15], [22], [26] were crossover design. The sample size ranged from 18 to 151. Six studies [14], [16], [21], [27] were made up of generally healthy populations; two studies [17] comprised of overweighed adults; the other fifteen studies included patients with ≥1 chronic conditions, such as dyslipidemia [15], [19], [26], T2DM [13], [22], [23], systemic lupus erythematosus (SLE) [20], peripheral artery disease (PAD) [18], human immunodeficiency virus (HIV) infection [24], acute myocardial infarction (AMI) [25] and chronic heart failure (CHF) [28]. The mean ages of the enrolled subjects ranged from 14 to 66 years old. The mean BMI ranged from 21 to 34.1 kg/m^2^. The mean SBP and DBP varied from 110.3 to 139 mmHg and from 58 to 81.9 mmHg respectively. The mean baseline levels of TG and TC ranged form 1.05 to 2.52 mmol/L, and from 4.43 to 7.33 mmol/L. Ten studies included participants who were all [19], [25] or partially [13], [18], [22], [23], [27] on statins, while ten studies only included participants who were not [14], [16], [17], [20], [21], [24], [26]. Twelve studies included participants who were all [25], [28] or patially [13], [18], [19], [22], [23], [27] on ACEIs/ARBs, while ten studies only included participants who were not [14], [16], [17], [20], [21], [24], [26]. The baseline FMD value varied from −1.99% to 9.5% (the FMD value below zero indicating constricted brachial artery in response to ischemia-induced hyperemia [9]–[11]). The dose of fish oil (defined as total dose of DHA and EPA) adopted in the included studies varied from 450 to 4530 mg/d, with the follow-up duration ranging from 2 to 52 weeks. Concerning the technical aspects of the FMD measurement, the occlusion position were forearms in 20 studies [13]–[20], [22]–[27], and upper arms in the other 3 studies [21], [28]; the occlusion duration was 5 min in 17 studies [13], [15], [17]–[19], [21], [23], [24], [26]–[28], 4.5 min in 4 studies [14], [20], [22] and 3 min in the rest 2 studies [16], [25].

### Data quality

The quality scores of the 23 studies ranged form 2 to 5. All of the included studies were randomized and controlled trials, with 19 studies in a double-blind design [13]–[15], [17], [20]–[24], [26]–[28]. Only 6 studies reported the method of random sequence generation [23], [27], [28], and 11 reported allocation concealment [14], [20], [22]–[24], [27], [28]. Details of withdrawals were reported in all of the included studies.

### Effects of fish oil supplementation on FMD

FMD was determined in all of the included studies by non-invasive ultrasound assessment of brachial artery endothelial responsiveness. The percentage change between baseline and endpoint induced by fish oil supplementation was used as the primary outcome. After data extraction and pooling, the meta-analysis was performed. The results of the pooled estimation revealed that fish oil significantly improved endothelial function in the included subjects, as demonstrated by increase of FMD (23 studies, 1385 subjects; WMD: 1.49%, 95% CI 0.48% to 2.50%, p = 0.004; **Figure** **2**). However, significant heterogeneity existed in terms of fish oil supplementation-related improvements of FMD (I^2^ = 87%, p<0.001).

### Meta-regression and subgroup analyses

Because differences in characteristics of participants and studies may contribute to the heterogeneity among the studies, we performed meta-regression analysis to explore the relationship between these study characteristics and the mean change in FMD after fish oil supplementation. The results of this meta-regression analysis revealed that among predefined variables (including number of the subjects in each study, mean age, BMI, SBP, DBP, TG, TC, mean baseline FMD of the participants, percentages of males, smokers and diabetic patients, dose of fish oil, EPA and DHA, follow-up duration and Jadad Score), Jadad Scores of the included studies were inversely associated with the FMD improvement after fish oil supplementation (regression coefficient  = −1.60, p = 0.04; **Table** **3**), indicating study quality may influence the overall effect of fish oil supplementation on FMD.

Subsequently, we performed subgroup analyses to evaluate how these predefined study characteristics may influence the effect of fish oil on endothelial function, as measured by FMD of brachial artery. The results of this analysis were similar to those in the meta-regression analysis described above. FMD improved significantly in studies of which the Jadad Score was 2 points (WMD: 8.99%, 95% CI 2.59% to 15.40%, p = 0.006; **Table** **4**), but didn't in studies of which the Jadad Score were 3 points (WMD: 0.65%, 95% CI −0.63% to 1.94%, p = 0.32), 4 points (WMD: 1.07%, 95% CI −1.32% to 3.46%, p = 0.38) or 5 points (WMD: −0.22%, 95% CI −0.75% to 0.30%, p = 0. 40). Besides, pooled analysis of studies including only normoglycemic participants showed significant improvements in FMD compared with those including only diabetic patients (p = 0.04; **Table** **4**); also, subjects with mean baseline DBP <75 mmHg seemed to experience more remarkable improvement of FMD than those with mean baseline DBP ≥75 mmHg (p = 0.01; **Table** **4**). Sensitivity analysis by including only double-blind, placebo-controlled studies also showed that fish oil supplementation didn't significantly influence the value of FMD based on these high-quality studies (19 studies, 1227 subjects; WMD: 0.54%, 95% CI −0.25% to 1.33%, p = 0.18; **Table** **4**), suggesting that pooled results derived from relatively high quality studies seemed not support a role of fish oil supplementation on improvement of FMD.

### Publication bias

The funnel plot for the effect of fish oil supplementation on brachial FMD was asymmetrical, suggesting the presence of publication bias (**Figure** **3**). Egger's significance test also indicated the existence of publication bias (p = 0.009). However, the result of the fail-safe N test indicated that it would take 266 unpublished null results (for all studies) to bring the combined p value to a nonsignificant level.

## Discussion

In this study, by pooling the results of the available randomized controlled trials, we found that fish oil supplementation significantly improved endothelial function, as measured by FMD. However, the results of meta-regression and subgroup analyses suggested the quality of the included studies (evaluated by Jadad Score) may influence the effect of fish oil supplementation on FMD, and the significance of the results seemed to depend mainly on the contribution of the studies with relatively poor quality (Jadad Score <3). Furthermore, complication of T2DM and baseline DBP of the included subjects may also influence the potential effects of fish oil on FMD. Normoglycemic participants and subjects with lower DBP (<75 mmHg) seemed to be associated with significant improvements of FMD after fish oil supplementation, while FMD of diabetic patients or the subjects with higher DBP didn't seem to benefit from fish oil supplementation.

Endothelial dysfunction has been recognized as an early pathophysiologic event during a variety of CVD, including hypertension and atherosclerosis [11]. More importantly, evidence from epidemiological studies and other clinical trials has suggested that impairment of endothelial function, as presented as a reduction of FMD, was an independent predictor for the incidence of cardiovascular events. Results of an early meta-regression analysis of 399 populations indicated that in populations at low CVD risk, endothelial function measured by FMD is related to principal cardiovascular risk factors, and to the estimated 10-year risk of coronary heart disease [5]. This is further supported by the evidence from a large population-based cohort study [6], which also found that brachial FMD is a predictor of incident cardiovascular events in multi-ethnic adults free of CVD, and may be of use to further improve the classification of subjects at low, intermediate and high CVD risk in addition to Framingham Risk Score. Besides, a recent meta-analysis [7], which mainly included studies in patients with established CVD, showed that impairment of FMD is significantly associated with future cardiovascular events and 1% reduction in brachial FMD was associated with 13% increase in risk of future cardiovascular events. In view of the above evidence, the result of our meta-analysis, which revealed that fish oil supplementation was associated with improvement of FMD by 1.49%, may be clinically relevant, indicating that the observed favorable effects of fish oil in primary and secondary prevention of CVD may be at least partially related to its effect on restoration of the endothelial function. Possible mechanisms underlying the beneficial effect of fish oil on endothelial function may include increase of membrane fluidity of endothelial cells, anti-inflammation, inhibition of platelets adhesion and aggregation, although the exact mechanisms involved are still unknown [3], [34].

Recent published meta-analysis by Wang et al [35] concerning the similar topic found that supplementation of omega 3 fatty acids significantly improves FMD by 2.30% and this effect may be modified by the health status of the participants or the dose of supplementation. Their study [35] is different from ours because besides studies with EPA and DHA, studies with another type of omega-3 fatty acids, alpha-linolenic acid, were also included. On the other hand, our study mainly focused on the effect of fish oil supplementation on FMD, and included some studies which have not been included in the meta-analysis by Wang et al [17], [24], [28]. Furthermore, we collected more detailed information of the baseline characteristics (particularly of the CVD risk factors) and background medication, which enabled us to further explore whether the difference of these factors were potential source of heterogeneity.

However, the results of our pooled analysis concerning the effect of fish oil supplementation on FMD should be interpreted with caution, because according to the results of the meta-regression and subgroup analyses, quality of the included studies may influence the overall effect, and the significance of fish oil's benefit on FMD was mainly driven by the studies of relatively poor quality. Sensitivity analysis by pooling only double-blind, placebo-controlled studies found that fish oil supplementation had no significant effect on FMD. This discrepancy may highlight the need of high-quality large scale trials in the future to evaluate the exact effect of fish oil supplementation on FMD and other markers of endothelial function.

Besides, we also found that complication of T2DM and levels of DBP of the included participants may influence the effects of fish oil on FMD. Specifically, FMD of participants with normal glucose metabolism or lower DBP seemed to be improved after fish oil supplementation, while those of diabetic participants or higher DBP didn't. The mechanisms underlying the above results were not known. In our opinions, it's possible that these results suggested that fish oil supplementation could only improve FMD in lower risk participants whose vascular function was largely preserved. This is because FMD is a biological process not only dependent on endothelial function (e.g. synthesis and release of NO and other endothelial derived vasodilators), but also dependent on the ability of vascular smooth muscle cells (SMC) to relax in response to the aforementioned vasodilators [9], [11]. Many factors (such as hyperglycemia [36], hypertension [37] and insulin resistance [38]) may contribute to the injury of vascular SMCs and subsequently impair their relaxation ability. In these circumstances, even though the endothelial function can be restored, FMD may not be improved. However, this hypothesis needs to be further tested in future studies.

A few recently published large-scale clinical trials [39], [40] and meta-analysis [41] failed to show a favorable effect of omega 3 fatty acids supplementation on cardiovascular events in patients who were of high risk or already with established CVD. It was suggested that perhaps the cardiovascular benefit of omega 3 fatty acids is limited with the improvements in cardioprotective drug treatment, such as use of statins and ACEIs/ARBs [42]. Because these two categories of drugs have been indicated to improve the endothelial function in former studies [43], [44], we investigated whether background cardiovascular therapy with statins or ACEIs/ARBs could impact the effect of fish oil on FMD. However, since detailed information (such as percentage of participants used, types of medication, and the number of participants who were on medication of target dose)of statins or ACEIs/ARBs usage are generally lacking in the included studies, we had to categorize these studies according to whether all, partial or none of the participants were on statins or ACEIs/ARBs therapy. Results of the subgroup analyses didn't suggest different effect of fish oil on FMD according to whether participants on statins or ACEIs/ARBs were included or not. Obviously, we couldn't conclude that background statins or ACEIs/ARBs usage didn't influence the effect of fish oil on FMD based on the above analyses, and these results should be interpreted very cautiously.

Our meta-analysis has several limitations. First, great heterogeneity was found among the included studies. Although we tried our best to include potential variables of study characteristics into our meta-regression and subgroup analyses, the heterogeneity couldn't be completely explained by these factors. Because we included subjects with substantial clinical heterogeneity, some other factors, including the baseline status of omega-3 fatty acids and concurrent medicines (including statins, ACEIs/ARBs and many other medications which may affect endothelial function) may contribute to the heterogeneity among the studies. However, details of these variables were generally not accessible in the included studies and therefore couldn't be analyzed intensively. Second, the number of included studies and the total number of subjects in some subgroup analysis (e.g. the diabetic subgroup only included 4 studies with 199 patients) were relatively small. Therefore, interpretation the results of the subgroup analyses should be with caution. Third, publication bias was found for the current meta-analysis, although the fail-safe N test suggested that it would take over 200 unpublished null results to bring the combined p value to a nonsignificant level. Finally, FMD was determined in the included study using non-invasive methods, which may not fully represent endothelial function, especially in subjects with higher CVD risk. Studies with other reliable markers of endothelial function may be needed to evaluate the effect of fish oil supplementation.

In conclusion, results of our meta-analysis indicated that fish oil supplementation significantly improved endothelial function, as measured by FMD. However, these results seemed to be mainly driven by the studies with relatively poor quality. High quality large scale clinical trials with adequate statistical power are needed in the future to further evaluate the effect of fish oil supplementation on endothelial function in the context of optimized concurrent cardiovascular therapy before we can come to a definite conclusion, especially in patients with high risk of CVD.
